# Sexual minority young adults’ perspectives on how minority stress and other factors negatively affect self-esteem: a qualitative interview study

**DOI:** 10.1080/09540261.2022.2051444

**Published:** 2022-04-25

**Authors:** Livia Bridge, Patrick Smith, Katharine A. Rimes

**Affiliations:** Institute of Psychiatry, Psychology & Neuroscience, King’s College London, London, UK

**Keywords:** Self-worth, youth, minority stress, qualitative

## Abstract

Sexual minority young adults (lesbian, gay and bisexual), are at increased risk of experiencing mental health problems than their heterosexual peers. On average they also have lower self-esteem which may contribute to the development or maintenance of mental illnesses. Interventions to improve self-esteem could improve well-being and reduce mental ill-health risk in sexual minority young adults. It is important to understand the processes that contribute to lower self-esteem in this population. The present study aimed to explore these processes. Semi-structured qualitative interviews were conducted with a sample of 20 sexual minority young adults (age 16–24 years) with a range of self-esteem levels. Using thematic analysis, three overarching areas were idenitified: ’Negative social evaluations and reduced belonging’, ‘Striving and failing to meet standards’, and ‘Negative sexual orientation processes’. These findings have theoretical implications for minority stress models of mental health inequalities, highlighting the potential interaction between minority-specific and more general risk factors for mental health problems. Findings also have clinical implications for the development of tailored interventions to help improve low self-esteem in sexual minority young adults.

Sexual minority young adults, for example who are lesbian, gay or bisexual, are more likely to experience mental health problems than their heterosexual counterparts (Birkett et al., 2015). Minority stress theories suggest that additional stressors due to stigma and discrimination epxlain this disparity. They also propose that self-esteem is one potential mediator that might partly explain the relationship between minority stress and mental health (Hatzenbuehler, 2009). There is evidence consistent with this suggestion (Oginni et al., 2019). Further, a recent meta-analysis found that self-esteem is, on average, significantly lower in sexual minority young people compared to heterosexual young people (Bridge et al., 2019). Not surprisingly, self-esteem is therefore a potential target for interventions to improve psychological well-being and mental health outcomes in sexual minority young adults. Understanding the processes that lower self-esteem in sexual minority young adults might help to develop tailored interventions aimed at improving self-esteem in this population.

In addition to minority stress, sexual minority young adults’ self-esteem might also be impacted by general stressors. Several longitudinal studies and meta-analyses have shown that self-esteem is most changeable in the transition from adolescence to young adulthood (Erol & Orth, 2011; Huang, 2010; Orth et al., 2010; 2018). Several important but potentially stressful developmental milestones take place for many young adults during this time and changes in self-esteem have been attributed to significant life-changes and challenges (Chung et al., 2014; Wagner et al., 2014). It is unclear what impact general stressors have on self-esteem in conjunction with experiences of minority stress.

The aim of the current study was to explore in detail the processes through which sexual minority young adults perceive that minority stress and other experiences have negatively affected their self-esteem. An explorative qualitative design was employed here given that no previous studies have examined the phenomenology of how minority stress or more general processes affects sexual minority young adults’ self-esteem.

## Method

### Participants

Participants were aged between 16–24 years and self-identified as a non-heterosexual sexual orientation (e.g. gay, lesbian, bisexual, pansexual, queer etc.). People were not included if they were currently experiencing a serious mental illness (e.g. anorexia, bipolar disorder, psychosis etc.) or were considered at high risk of suicidality. This was due the possibility that the interview may involve discussion of distressing memories. Recruitment continued until theme saturation was reached, resulting in a sample of twenty participants. This study was part of a larger qualitative project with the same participants, which also looked to identify potential protective and coping factors for self-esteem.

### Procedure

Participants were recruited using purposive sampling and efforts were made to recruit sexual minority young adults with a range of different self-esteem levels. The study was primarily advertised online through a UK research participation recruitment website and on social media. Interested participants were sent an information sheet and then invited to a telephone screening call if still interested. Eligibility was checked during telephone screening and provided a chance for participants to ask questions. Eligible participants were invited to an interview at this point. Informed consent was then obtained, and participants completed questionnaire measures online before interview.

Interviews were conducted by the first author. Interviews were audio-recorded with the consent of participants. Participants received token payment as compensation for their time and travel. Ethical approval for this study was granted by the relevant research ethics subcommittee at King’s College London (Ref: HR-17/18-5266). Each interview followed a semi-structured interview guide outlining broad questions to be explored with suggested prompts (see Table 1). Questions in the interview guide were developed based on a literature review and gaps within the literature, with feedback from sexual minority young people. Open ended and flexible questions allowed participants to focus on experiences that were important to them.

**Table 1.** Interview guide questions and prompts.

Question	Prompts
Please could you tell me about your self-esteem?	Level Impact on life Changes over time
What are the most important factors/experiences that you think have had a negative impact on your self-esteem?	Could you tell me more about how they have affected your self-esteem? Relationships Stressful life events Family
[if not mentioned already] Have you experienced stigma or discrimination from others related to your sexual orientation?	Could you tell me a bit more about this? From who? Direct/indirect Microaggressions
Have these experiences of stigma or discrimination affected your self-esteem or how you feel about yourself?	[If yes]: Could you tell me about how this affected your self-esteem? Thoughts/feelings Activities Relationships

### Measures

Measures of self-esteem, anxiety, depression, and self-criticism were collected before each interview to help describe the psychological characteristics of the sample.

### Rosenberg Self-Esteem scale (RSES)

The RSES is a 10-item self-report measure of global self-esteem. Statements are rated for level of agreement on a 4-point scale ranging from ‘Strongly agree’ to ‘Strongly disagree’. Good internal consistency was found for this sample (alpha= 0.83). Total scores range from 10–40; higher scores represent higher self-esteem. A score <25 indicates clinically low self-esteem (Rosenberg, 1965; Isomaa et al., 2013).

### Patient health questionnaire (PHQ-9)

The PHQ-9 is a 9 item self-report measure of recent depression symptoms. Frequency with which symptoms have been experienced over the last 2 weeks are rated on a 4-point scale ranging from ‘Not at all’ to ‘Nearly every day’. A total score is calculated out of 27; higher scores indicated more severe depression. The PHQ-9 has demonstrated good internal consistency in normative samples, ranging from 0.86 to 0.89 (Kroenke et al., 2001).

### Generalised anxiety disorder questionnaire (GAD-7)

The GAD-7 is a 7-item self-report measure of recent anxiety symptoms. Participants rate the frequency with which they have experienced symptoms over the last two week on a 4-point scale ranging from ‘Not at all’ to ‘Nearly every day’. Scores can range from 0–21; higher scores indicate more severe anxiety. Previous studies have shown the GAD-7 to have good internal consistency (Cronbach’s alpha = 0.92) (Spitzer et al., 2006).

### Forms of Self-Criticising/attacking & Self-Reassuring scale (FSCRS)

The FSCRS is a 22 self-report measure of different forms of self-critical and self-reassuring responses to a mistake or setback. Three sub = scales included are: ‘inadequate self’ (9 items; internal consistency = 0.75), ‘hated self’ (5 items; internal consistency = 0.86), and ‘self-reassurance’ (8 items; internal consistency = 0.86). Level of agreement with statements is rated on a 5-point Likert scale from ‘Not at all like me’ to ‘Extremely like me’ (Gilbert et al., 2004).

### Data analysis plan

A bracketing interview was conducted within the research team to understand and manage the interviewers’ assumptions before data collection. Analysis followed the Braun and Clarke (Braun & Clarke, 2006) six-step guidelines for thematic analysis. Interviews were audio-recorded and then transcribed verbatim by either the interviewer (first five) or a professional external transcription service. Line by line coding was then completed for the first five interviews. Initial codes were organised to create a ‘working thematic map’ consisting of potential themes and sub-themes. New codes identified from line-by-line coding of the remaining 15 interviews were added to the ‘thematic map’. Theme saturation was reached when no new codes appeared from interviews and recruitment stopped at this point. Higher-order themes were developed through an iterative process of new code identification and theme refinement. Themes were discussed within the research team several times during the refinement period to check their theoretical credibility. Final themes and sub-theme headings were agreed within the research team.

## Results

### Participant characteristics

Participant characteristics and scores on clinical measures are summarised in Table 2 below. Half of participants self-reported clinically low self-esteem on the Rosenberg self-esteem scale whilst all the young adults felt that they had low self-esteem at some point in their lives.

**Table 2.** Participant characteristics and scores on clinical measures.

Participant Characteristics		
Age (SD)	20.1 (2.3)	
Gender identity	*N* (%)	
Man (Cisgender)	7 (35)	
Woman (Cisgender)	12 (60)	
Non-binary	1 (5)	
Sexual Orientation		
Lesbian	6 (30)	
Gay	3 (15)	
Bisexual	7 (35)	
Pansexual	2 (10)	
Queer	1 (5)	
Asexual/Bi-romantic	1 (5)	
Ethnicity		
White British	6 (30)	
Black	3 (15)	
Asian	4 (20)	
Other White	3 (15)	
Mixed Background	4 (20)	
Measures	Mean (SD)	Range
RSES	26.1 (5.8)	18–33
FSCS		
IS	21.1 (9.2)	1–34
HS	16.2 (7.2)	0–15
RS	5.3 (4.7)	4–29
GAD-7	6.9 (4.4)	0–14
PHQ-9	7.4 (5.2)	0–17

RSES: Rosenberg Self-esteem Scale; FSCS: Forms of Self-criticising/attacking and Reassuring Scale (IS: inadequate self; HS: hated self; RS: reassuring self); GAD-7: Generalised Anxiety Disorder-7 Questionnaire; PHQ-9: Patient Health Quesitonnaire-9.

Note that quotes in themes are labelled with the associated participant number, their gender identity (i.e., M = man, F = woman, NB = non-binary) and sexual identity (G = gay, L = lesbian, B = bisexual, O = other sexual identity).

### Minority stress: experiences of stigma, prejudice, and discrimination

All participants reported that they had experienced sexual orientation-related stigma, discrimination, or stress. Minority stressors in this context reported here included societal stigma, microaggressions, being rejected by close others, homophobic bullying, physical abuse, and invalidation from within the LGBTQ + community itself. Some participants also reported minority stress stemming from the intersectionality between their sexual orientation and other minority characteristics. For example, several participants spoke about the stigma of their sexual orientation within their religious or ethnic community whilst others spoke about microagressions and stereotypes related to being a women and having a sexual minority orientation.

### General life stressors

All participants also discussed more general life stressors. These included traumas, separation of parents, physical illnesses, financial difficulties, caring for family members with mental health problems, bullying and exclusion by peers, and academic stressors (e.g. deadlines/starting university).

### Themes about processes contributing to lower self-esteem

Three key themes about the processes involved in the development and maintenance of lower self-esteem emerged from thematic analysis. These were: ‘Striving and failing to meet standards’, ‘Negative social evaluations and reduced belonging’, and ‘Negative sexual identity processes’. The major themes were interrelated and should not be considered as independent concepts. The three themes and their subthemes are described below.

### Negative social evaluations and reduced belonging

#### Perceived value and acceptance from others

All participants talked about the extent to which they felt valued by others as being central to their self-esteem. For example, one participant expressed that *‘for me the main thing to do with self-esteem is my social standing and other people’ views of me’ (MB5)* whilst another mentioned how this affected their feelings of worth because *‘a lot of my self-esteem is based off of how people feel about me and what I mean to them. So, if that’s affected, then that, in turn, makes me feel like I might not be worth it’ (FB9).* Feeling less valued socially could be preceded by either minority stress experiences or general negative events related to social standing or value.

Several participants mentioned that difficult romantic or family relationships had affected how valued they felt. One participant felt that, being neglected by their parents led them to lose self-worth *‘because I felt like my parents weren’t valuing me I lost my sense of worth really’ (FB4).* Feeling rejected by or unacceptable to friends and family because of their sexual attractions or identity affected young adults’ self-esteem as this related to value from **‘***people I care about***’.** Participants reported that although it didn’t necessarily lead to negative feelings about their own sexuality it made them *‘feel less good about yourself if you think that your parents aren’t going to love you as much as they did before’ (FL12). F*eeling less valued or loved therefore affected their general self-worth, as one participant mentioned:

*you want your parents’ approval and if you can’t get it then you maybe feel bad about yourself and so, feeling that something I couldn’t change wouldn’t be wholly approved of – they wouldn’t be actively hostile, I knew that, but that they wouldn’t be exactly pleased is something that will affect your self-worth (FL20)*

#### Not fitting in

Almost all participants expressed having felt *‘different’* or that they didn’t ‘*fit in*’ or *‘belong’* at some point in their lives. This affected their self-esteem when it was interpreted as evidence that there was *‘something wrong*’ with them or they were *‘not normal’.* The feeling of not fitting in could be related to minority stress experiences or other external factors such as race, religion or socioeconomic status and bullying or social exclusion. For other participants, feeling that they did not fit in was a general sense of low self-worth which could be related to their sexual orientation, rather than specific experiences e.g. *‘I’m not straight. So, it’s the combination of those makes me feel like an outsider. You’re going against what’s seen as average in society and that’s never going to be easy’* (FL19). Hiding their sexuality or being less open as a result of fear of a negative reaction also exacerbated this problem for some participants.

Several participants felt that their race or religion had affected how easy it was to interact with other people and had tried *‘to blend in as much as possible’ (FL18).* Not speaking the same language or being from the same culture as the majority led to difficulty finding a place where they felt they belonged and lowered their self-esteem. For several young adults who were bisexual, they mentioned that fitting in could be even more difficult, as they felt they experienced prejudice from within the LGBTQ + community, reducing their sense of belonging further, as discussed by the participant:

*I think actually being bi you can also get discrimination from the LGBT community, so I found it a lot harder to come out as bi to them. I’m quite wary of telling them that I’m bi rather than gay because I think that might change the relationship slightly. I feel a bit like a fraud it’s the whole not belonging in a place (FB4)*

#### Social withdrawal

Several participants reported *‘withdrawing’* from other people in response to low self-esteem and negative thoughts about themselves. Implications of withdrawal on mood, behaviour and relationships were then perceived to lower self-esteem further or maintain low self-esteem. For some young adults, they felt that lower self-esteem reduced their motivation to socialise. Social withdrawal was seen by some young adults as a coping mechanism to prevent rejection. Withdrawing from friends and family when self-esteem was low in turn prevented young adults from receiving the boost to self-esteem of positive relationships and spending time with others. This could become a vicious cycle where relationship quality was impaired, and this maintained low self-esteem and mood.

*I think it’s, like, mostly because of my self-esteem that my mood is, like, quite bad. I like to be alone, like, not talk to anyone … but then I feel very trapped – like, I want to disappear, in those situations I feel very worthless and really small (FB15)*

### Striving and failing to meet standards

#### High standards and self-criticism

Over half of participants mentioned that having high standards for themselves had negatively affected their self-esteem. Some young adults reported high expectations being intrinsic to themselves whilst others attributed this to the external standards from their environment, including highly critical parents. High expectations were often related to academic performance or careers, but several young adults also reported high standards in relation to their sexual orientation and being an advocate for sexual minority rights.

*I want a good job and I want to travel the world and I want – like certain life things. And I feel like if I’m not taking enough steps to achieve those, then that’s where my – where I’m shortcoming and that’s when my self-esteem is dipping. (FB10)*

High expectations were linked to self-criticism when they were not perceived to be met. Self-criticism included having thoughts that *‘beat’* themselves up or were very *‘harsh’* on themselves. Many participants spoke about criticism as being a pervasive response to perceived failures or mistakes, expressing that they tend to *‘criticise everything just everything’ (FB4) or* be their *‘own worst critic’ (MG11)*. Self-criticism was usually triggered as a response to a specific event but then generalised to overall value or self-esteem.

For several participants, self-criticism could also be a response to minority stress experiences. For example, one participant spoke about the impact that gender-nonconformity related homophobic bullying had on his subsequent self-criticism and self-esteem:

*When I moved to a different school I… was constantly evaluating myself, whether I’m not too girly … and very often being angry at myself and really hating my voice … it had rather a bad impact on my self-esteem. (MG16)*

#### Over-thinking: repetitive self-critical thinking

Several participants reported tending to ‘overthink’ or ‘dwell’ on negative experiences with a self-critical focus which in turn lowered their self-esteem. Overthinking could also be in response to minority stress experiences such as verbal abuse or societal stigma. For several of the young adults this started as angry rumination towards the external situation but then led to self-criticism and lower self-esteem as demonstrated in the quote below.

*Maybe I’m obsessing about this. Maybe this isn’t a big issue. Maybe this is just my insecurity. Why do I care so much about what other people think? Oh, God, I’m so, kind of, like, pathetic, and then suddenly, it’s back on that loop. (MO7)*

For several others, overthinking was reported as relating to their experiences of concealing their sexuality. For example, one participant discussed repeatedly thinking about whether *‘I shouldn’t’ve hidden this …. I should’ve said things or not said things’ (FB10).*

#### Social media standards, comparisons and body image/appearance concerns

Many participants reported that standards conveyed in social media had negatively affected their self-esteem. Exposure to sites presenting unrealistic body images or achievements had a negative impact on participants’ feelings about their own body, achievements, or life. Several young adults suggested this was linked to comparing themselves to other people to measure their own value or worth. Several participants mentioned that making upward *‘comparisons to other people being better’ (FL08)* then contributed to their negative view of themselves.

Body image and appearance concerns were the most frequently discussed consequence of social comparisons and half of participants did speak about standards relating to their body and appearance being strongly linked to self-esteem. This included self-esteem being poor through feeling ‘unattractive’, ‘ugly’ or ‘fat’ as well as more specific concerns about certain aspects of appearance such as height, skin, hair, or muscular tone. For some, their body image concerns were related to their sexuality or being part of the LGBTQ + community. One young adult who identified as non-binary spoke about *‘hating [their] body hair’,* being *‘too weedy’* and feeling quite *‘body dysmorphic’ (NB2)*, which related to their gender identity. Another who identified as pansexual discussed how *‘body image issues really did tie in with my sexuality, cause it wasn’t just, like, one standard that I was failing, it was, like, two or three’* (MO7*).* Body image issues related to sexuality were also discussed by several young lesbian women. For example, one participant spoke about an *‘absolutely entrenched femme privilege’ (FL8)* in society, and this caused her to feel less attractive within her more *‘butch’* lesbian relationship.

### Negative sexual identity processes

#### Shame and internalised homophobia/biphobia

Some participants reported negative beliefs about their own sexuality, a lack of self-acceptance, or *‘internalised homophobia’* resulting from minority stress experiences including bullying, microaggressions, societal stigma, or rejection by family/friends. This included feelings such as being *‘disgusted’* by their sexuality as well as thinking they were *‘divergent’*, ‘*dirty*’, *‘wrong’* or ‘*not normal*’ because of their sexual orientation. These more specific negative beliefs towards their sexuality then in turn affected their general self-worth, reporting feeling like an *‘awful’* or *‘bad person’* or *‘not liking’* and *‘hating’* themselves. As one participant explained**:** ‘[hearing about] *society having a certain stigma that being a certain way was wrong and that the sexual act was wrong – it didn’t make me feel very good about myself’ (MG11).*

For some, shame or negative beliefs, as well as being directly related to lower self-esteem, were linked to hiding their sexual identity, which inevitably affected self-esteem.

*I think, initially, I did feel a bit, sort of like, not disgusting, but like, not a very nice, sort of, person because of how people in society viewed, or certain people in society, viewed gay people. So, I really didn’t like myself and I felt like, you know, I’m disgusting, and I have to hide this for a really long time (MG11)*

#### Impact of hiding a stigmatised sexual orientation

Almost all participants discussed having hidden their sexual attractions, identity, or related behaviours from others because they feared negative reactions or damage to relationships by also changing their appearance or monitoring behaviour to hide their sexuality. This often involved trying to appear less gender non-conforming to fit in with gender norms associated with a heterosexual sexual orientation. Not being open or having to ‘hide’ their identity had a significant impact on how many participants viewed themselves, with one young adult, for example, mentioning that *‘self-esteem and closetedness go hand-in-hand’ (MO7***).**

Keeping sexual identity, or parts of it, hidden impacted young adults’ self-esteem in several ways. Firstly, some were self-critical of hiding and not being honest with other people. Secondly, several participants spoke about how hiding their sexuality from others led to thoughts that there is something wrong with it and a lack of self-acceptance.

*You have to have low self-esteem, really, to continue living being closeted and believing that what you are is inherently wrong. You can’t do that and have high self-esteem. There’s no way of not being okay with who you are and being okay with who you are. (MO7)*

Finally, not being able to be open about their romantic relationships and sexuality was damaging to relationships for some young adults. Concealment led to feelings that they didn’t ‘fit in’ or were different from others. Negative implications for their social relationships in turn had a negative impact on their self-esteem.

#### Sexual orientation uncertainty

Several participants reported feeling doubt, confusion, or uncertainty about their sexual identity at some point. Identity uncertainty and the impact on self-esteem was discussed as a result of experiences of bullying or homophobia e.g.: *‘if you’re unsure, like, already about yourself and how you’re feeling and you’re not quite sure what to make of it, then it’s harder to, like, obviously, raise your self-esteem, because you’re so confused’,* and microaggressions such as negative stereotypes (e.g. same sex attractions being a ‘phase**’**), for example, for one participant:

*it invalidates my sexuality to me, so, I’m suddenly thinking, right, I’m not being true to myself, but then I can’t work out if I am being true to myself or not … which is uncertainty that isn’t exactly a glowing self-esteem point (NB2).*

## Discussion

This qualitative interview study and thematic analysis highlights the interaction between sexuality specific and more general processes which sexual minority young adults perceive have negatively impacted their self-esteem. Our findings highlight the importance of general life stressors and their impact in addition to sexual minority specific processes on the self-esteem of sexual minority young adults.

Negative interpersonal experiences and a reduced sense of belonging, both sexuality-related and not, were perceived by participants to impact self-esteem through a lack of perceived value to others. This is consistent with social theories of self-esteem development and maintenance (Leary, 2003a, 2005). Sociometer theory proposes that self-esteem is an internal monitor of how valued or accepted we feel by others. Findings here demonstrated that minority stress experiences might partly impact self-esteem through this process. Sexual minority young adults in the current sample described how perceived rejection or non-acceptance of their sexual orientation had negatively affected their self-esteem whilst not necessarily their positivity about their own sexual identity.

The second theme identified here potentially relates to the transdiagnostic concept of self-critical or ‘clinical’ perfectionism and is an example of ‘dysfunctional’ or’ unhelpful assumptions’. Shafran, Cooper & Fairburn’s cognitive behavioural model of clinical perfectionism suggests that self-criticism after failure to meet standards is a key process that maintains self-evaluation that is overly dependent on meeting personal standards and may result in anxiety and depression (Shafran et al., 2002). Research has found consistent associations between this type of perfectionism, increased emotional distress, and lower self-esteem (Egan et al., 2011). The current study highlights how perfectionism might also extend to experiences of minority stress. Sexual minority young adults in the current study reported having self-critical thoughts and in turn lower self-esteem, after perceived failure to respond to sexual minority prejudice, or discrimination, in a way that met their standards.

Current findings also support previous research which has consistently found that body image dissatisfaction predicts lower self-esteem in adolescence and emerging adulthood within the general population (Tiggemann, 2005; von Soest et al., 2016). Appearance is one domain where previous research has found that young gay men place more contingency for their self-worth compared to heterosexual young men (Pachankis & Hatzenbuehler, 2013). Body dissatisfaction and eating disorders are also more prevalent in gay young men compared to heterosexual young men and there is evidence that body dissatisfaction is associated with lower self-esteem in this population in both directions (Boroughs et al., 2010; Gil, 2007; McArdle & Hill, 2009). The current study findings suggest that self-esteem might be adversely affected by perceived ideals related to body image in sexual minority individuals with other gender and sexual identities too. For example, young women in this study discussed concern over the ideal of appearing more ‘femme’ as a lesbian young woman. For polysexual identities, where young adults here reported a perceived failure to meet both heterosexual and LGBTQ + appearance standards, the impact on self-esteem could be twofold. The role of social media in promoting unrealistic body image ideals was highlighted by participants in this study.

Not surprisingly some sexual minority young adults in this study also expressed how their self-esteem had been impacted by negative thoughts or behaviours related to their sexual identity. Current findings are consistent with previous cross-sectional evidence demonstrating that internalised homophobia may mediate the relationship between external minority stress experiences and self-esteem (Blais et al., 2014; Martin-Storey & Crosnoe, 2012). Young adults here expressed how they felt that internalisation of negative beliefs about their sexual identity in turn generalised to global self-criticism and feelings of low self-worth. This evidence is in line with findings from previous qualitative research exploring the impact of parental rejection on self-worth (Carastathis et al., 2017), but extends this to other forms of minority stress.

Current findings also highlight how concealment behaviours intended to prevent rejection can have a strong negative impact on self-esteem for sexual minority young adults. A cognitive-affective-behavioural model has previously been proposed to explain the potential negative impact of concealing a stigmatised identity on self-evaluation (Pachankis, 2007).

The current study provides support and additional detail for several of the mechanisms proposed in the model. Hiding behaviours were felt by some young adults here to have impaired the quality of their social relationships and therefore reinforced beliefs that they don’t ‘fit in’. Hiding also appeared to prevent positive sexual identity development for some young adults; where hiding their sexuality fed into the feelings of shame and negative beliefs they felt about their own sexuality. Some young adults also appeared to hold themselves to very high standards for being open about their sexuality, resulting in self-criticism (e.g. being a fraud or liar) and reduced self-esteem. These cognitions are in line with previous research demonstrating that feeling like one’s authentic self is important for self-esteem in order to feel genuinely valued by other people (Leary, 2003b; Riggle et al., 2017).

Sexual orientation uncertainty was one sexual identity specific process reported here as having negatively impacted self-esteem that has not routinely been included in minority stress models. Participants discussed being critical of themselves for not being sure about their sexual attractions or identity. Uncertainty also appeared to prevent some young people from forming more positive self-beliefs around their sexual orientation. Lower self-esteem might therefore help partly explain findings from previous studies that adolescents who report higher levels of identity uncertainty experience higher rates of psychological distress or negative mental health outcomes (Dyar et al., 2015).

## Limitations

The interpretations and conclusions that can be drawn from findings are limited due to the subjective and constructivist nature of both semi-structured interviews and thematic analysis. Further, generalisability of findings should be cautious due to some limitations of the sample. Although participants identified with a range of different sexual orientations and ethnicities, the highest percentage were still white and almost all lived in a large urban environment. Participants were also not directly asked about the reciprocal impact of self-esteem on their stigma-related experiences or responses. Relative levels of self-esteem might influence perception of and responses to minority stress experiences. People may be unaware of how they might behave or think differently if their level of self-esteem were different.

## Theoretical and clinical implications

Findings here have potential implications for minority stress models and theoretical perspectives on the function of self-esteem. Transdiagnostic processes identified here including self-critical perfectionism, self-critical rumination, and social withdrawal, might be added to Hatzenbuehler’s (2009) psychological mediation framework which suggests that sexual minority stigma can impact on wellbeing via general psychological mechanisms, such as lower self-esteem, as well as sexual minority-specific processes. The interaction between self-esteem and sexual minority specific processes such as internalised stigma and sexual orientation concealment is also supported by current findings and sexual orientation uncertainty is an additional process identified here that might be added to models. The significant impact reported by the young adults here of social relationships and perceived value or acceptance on their self-esteem, supports proposals from social evolutionary theories that self-esteem serves as an internal subjective monitor of our value in relation to others. Our findings suggest that psychological interventions aimed at ameliorating low self-esteem in sexual minority young adults should focus on both sexual minority specific and general processes.
